# Failure Analysis of Ultra High-Performance Fiber-Reinforced Concrete Structures Enhanced with Nanomaterials by Using a Diffuse Cohesive Interface Approach

**DOI:** 10.3390/nano10091792

**Published:** 2020-09-09

**Authors:** Umberto De Maio, Nicholas Fantuzzi, Fabrizio Greco, Lorenzo Leonetti, Andrea Pranno

**Affiliations:** 1 Department of Civil Engineering, University of Calabria, 87036 Rende, Italy; umberto.demaio@unical.it (U.D.M.); lorenzo.leonetti@unical.it (L.L.); andrea.pranno@unical.it (A.P.); 2 Department of Civil, Chemical, Environmental and Materials Engineering, University of Bologna, 40136 Bologna, Italy; nicholas.fantuzzi@unibo.it

**Keywords:** ultra high-performance fiber-reinforced concrete (UHPFRC), nanomaterials, diffuse cohesive interface models, nonlinear finite element analysis, multiple crack propagation

## Abstract

Recent progresses in nanotechnology have clearly shown that the incorporation of nanomaterials within concrete elements leads to a sensible increase in strength and toughness, especially if used in combination with randomly distributed short fiber reinforcements, as for ultra high-performance fiber-reinforced concrete (UHPFRC). Current damage models often are not able to accurately predict the development of diffuse micro/macro-crack patterns which are typical for such concrete structures. In this work, a diffuse cohesive interface approach is proposed to predict the structural response of UHPFRC structures enhanced with embedded nanomaterials. According to this approach, all the internal mesh boundaries are regarded as potential crack segments, modeled as cohesive interfaces equipped with a mixed-mode traction-separation law suitably calibrated to account for the toughening effect of nano-reinforcements. The proposed fracture model has been firstly validated by comparing the failure simulation results of UHPFRC specimens containing different fractions of graphite nanoplatelets with the available experimental data. Subsequently, such a model, combined with an embedded truss model to simulate the concrete/steel rebars interaction, has been used for predicting the load-carrying capacity of steel bar-reinforced UHPFRC elements enhanced with nanoplatelets. The numerical outcomes have shown the reliability of the proposed model, also highlighting the role of the nano-reinforcement in the crack width control.

## 1. Introduction

Nowadays, concrete results in being the most used construction material in building and civil engineering, due to its durability, affordability, high temperature resistance, and good compressive strength [1]. Its limitations, such as quasi-brittle behavior and low tensile strength, have motivated several researchers to invest time and resources in the design and production of new advanced concretes. In particular, the so-called ultra-high performance concrete (UHPC), characterized by high cement content, small aggregate size, high binder content (fly ash, silica fume, reactive powder, etc.), and low water/cement ratio (<0.2), has been developed to achieve enhanced mechanical properties respect to the ordinary concrete, in terms of both compressive strength (up to 100–200 MPa) and tensile strength (up to 5–10 MPa) [2,3,4].

In general, UHPC exhibits an increasing brittleness with the increase of compressive strength, but such a brittleness can be properly limited with the inclusion of discontinuous high-strength steel fibers. This addition is able to enhance both the ductility and the flexural strength of concrete [5,6,7], and it leads to the definition of the so-called ultra high-performance fiber-reinforced concrete (UHPFRC). Depending on the shape and content of embedded fibers, UHPFRC exhibits different mechanical behaviors. Specifically, by using undeformed or deformed (for instance hooked-end, twisted or wave-shaped) steel fibers, a strain-softening or strain-hardening behavior can be typically obtained, respectively [8,9]. Several experimental studies about the effects of both the length and amount of steel fibers on the global structural response of cementitious matrices have clearly shown that, by increasing the fiber volume fraction, a sensible performance improvement in terms of both tensile strength and ductility can be obtained [10,11,12,13]. In particular, with the incorporation of 1%, 2%, and 3% of straight steel fibers in UHPC samples, their flexural strength significantly increases by about 21%, 47%, and 100%, respectively [14]. In addition, as observed in [15], the UHPC tensile strength results in being 60% higher by using deformed steel fibers compared to straight steel fibers, and the tensile strain measured at the peak stress is around three times higher.

Despite the excellent mechanical properties offered by UHPFRCs, previous studies indicate that their flowability is negatively influenced by high values of the fiber content [16,17] and thus, in order to develop advanced concretes with an improved workability, new kinds of reinforcement, involving different scales of observation (i.e., nano- and micro-scales) in the same mixture, have been recently investigated [18,19,20,21]. Specifically, it has been demonstrated that the addition of only micro-reinforcement in UHPFRC results in being scarcely effective in delaying fracture onset and propagation phenomena, while recent studies in nanotechnology have highlighted the capability of nano-sized reinforcement to enhance the performances of cement-based materials in terms of both strength and fracture toughness [22,23,24] without any substantial reduction in their workability. Currently, several types of nanomaterials have been used as a reinforcement of the cementitious paste, such as nanosilica, nanoiron, and, more recently, carbon nanotubes (CNTs) and graphene sheets [25,26,27]. Among these, carbon-based nanomaterials, which are available in different shapes (particles, fibers, sheets and platelets), possess the best mechanical properties in terms of elastic modulus (reaching values of about 1000 GPa) and tensile strength (with values greater than 100–200 GPa) [28,29,30,31,32].

Definitively, the improved flexural and compressive strength and fracture toughness properties of concrete provided by the nano-sized reinforcement, used in combination with other reinforcement types, led to the development of a new-generation concrete, consisting of a special UHPFRC enhanced with nanomaterials. However, despite extensive studies on the influence of nanomaterials on the mechanical properties of concrete and mortar are available in the literature, only a few experimental investigations on UHPFRCs enhanced with nanomaterials have been performed. Among these, some authors showed that, with a 0.3% of graphite nanoplatelets embedded in a UHPFRC element, an increase of 56% and 187% in the tensile strength and energy absorption capacity can be obtained, respectively [33]. Other researchers investigated the role of embedded nanomaterials on the bond behavior between steel fibers and UHPC by means of pull-out tests, showing that a significant increase in the bond stresses can be achieved with the addition of only the 0.02% of carbon nanotubes [34]. Moreover, some authors identified the optimal amount of combined steel microfibers and carbon nanofibers embedded in a UHPFRC providing a balanced increase in the flexural and compressive strengths [35]. Furthermore, a significant increase in the bond between cement matrix and steel microfibers is obtained in [36], via the incorporation of a small percentage of nanotubes, thus leading to a higher concrete pull-out strength. Specifically, the above-mentioned enhanced bond strength, associated with a stronger crack bridging effect in the proximity of the steel fibers, is capable to inhibit the micro-crack onset and to delay the subsequent fracture propagation processes.

Despite the great number of experimental studies, to the best knowledge of the authors, only a few works in the literature have been addressed to the investigation of nano-enhanced UHPFRCs from the computational point of view, essentially due to the severe separation of the spatial scales involved in the damage and/or fracture phenomena, resulting in the difficulty of incorporating the toughening effect of nano-reinforcement in the overall (i.e., macro-scale) constitutive behavior of concrete (see, for instance, [37]).

More generally speaking, cracking processes in fiber-reinforced concretes (FRCs), mainly without nano-enhancements, have been simulated in the literature by means of a wide variety of numerical models and methods, based on either smeared or discrete fracture approaches, in which the improved ductility provided by the dispersed reinforcement has been accounted for by properly modifying the softening behavior of the adopted (bulk or interface) constitutive models.

Smeared crack approaches, consisting of smearing any individual crack over a computational region with degraded material properties, work naturally with the continuum mechanics-based computational methods, such as the standard displacement-type finite element method, but inevitably lose the discrete nature of fracture. Among the existing smeared approaches, damage, plasticity, and combined damage/plasticity models have been successfully applied to FRC materials. These models usually adopt different regularization techniques able to prevent the well-known ill-posedness of the associated BVPs, such as those based on strain gradient [38] and micropolar [39] formulations. In particular, the popular Concrete Damaged Plasticity (CDP) model has been adopted, with or without ad-hoc modifications in its constitutive formulation, for the failure simulation of UHPFRCs (see, [40,41], respectively). It is worth noting that most of the available continuum damage models for UHPFRC are of a phenomenological type, but there exist some sophisticated modeling approaches which consider UHPFRC as a two-phase composite with different constitutive behaviors of cement matrix and short fibers, combined together in a single microscopically derived anisotropic damage model for such a material [42].

Instead of modeling cracks in a smeared manner, discrete fracture approaches possess the advantage of describing cracks as individual discontinuity surfaces embedded in the computational domain, thus taking into account, in a detailed way, multiple crack initiation and propagation, crack coalescence, unilateral contact between crack faces with or without friction, debonding and other interacting nonlinear micro- or macro-scale phenomena occurring not only in cement conglomerates, but also in other brittle and ductile reinforced composite materials (see, for instance, [43,44,45,46]).

Among the existing discrete fracture approaches, cohesive zone models (CZMs) are the most used for concrete-like materials, including FRCs. In this context, a variety of cohesive traction-separation laws have been proposed, able to account for the influence of embedded short fibers on the global fracture properties (flexural strength, tensile fracture energy, etc.) of the resulting concrete [47,48,49]. In particular, a simple and practical trilinear softening model for FRCs has been proposed in [48], based on the separation of related fracture mechanisms into the aggregate bridging zone and the fiber bridging zone. Most of the existing cohesive zone models for FRCs have been derived in a phenomenological manner, but, recently, some interesting multiscale fracture approaches have been proposed, in which the individual nonlinear behaviors of concrete matrix and short fibers, as well as their mutual interactions involving different length scales, have been accounted for in the interfacial constitutive modeling of FRCs at the macroscopic scale (see, for instance, [50]).

From the analysis of the current technical literature, it can be observed that the attention is mainly focused on the numerical simulation of simple laboratory-scale UHPFRC elements without considering accurately the additional strengthening effect of steel reinforcing bars required for real-life engineering applications. Indeed, a comprehensive computational framework able to accurately predict all the potential damage mechanisms in steel bar-reinforced nano-enhanced UHPFRC structures under both ultimate and service loading conditions seems to still be missing, probably due to the inherent difficulty of incorporating all the different steel/concrete interactions in the already complex constitutive behavior of damaged concrete with both micro- and nano-reinforcements.

The present work may be regarded as an attempt to fill this gap, by proposing a novel integrated numerical model for steel bar-reinforced UHPFRC structures, including those enhanced with the incorporation of nanomaterials. The two main ingredients of this model, detailed in Section 3, are:

- a diffuse interface modeling (DIM) approach, based on a cohesive finite element methodology, introduced by some of the authors in [51] for conventional concrete and here adapted to the case of nano-enhanced UHPFRC. This approach, whose theoretical background is briefly recalled in Section 2, considers all the internal mesh boundaries as potential crack segments, modeled as cohesive interfaces equipped with a suitably calibrated mixed-mode traction-separation law. The desired feature of this approach consists in the possibility to simulate multiple crack onset and propagation without requiring externally introduced crack initiation criteria or computationally costly remeshing operations;
- an embedded truss model (ETM), used in synergy with a special sliding interface model, already adopted in [52,53] for conventional RC structures and here adapted to capture the enhanced steel/concrete bond-slip behavior provided by nano-reinforcements embedded in the UHPFRC mixture.

In the first part of the results (Section 4), the main applications of the proposed fracture approach to plain nano-enhanced UHPFRC are presented for both validation and parameter calibration purposes. Suitable comparisons with the available experimental results, involving a four-point bending test on small-sized UHPFRC beams with different volume fractions of graphite nanoplatelets (GNPs), are provided to show the accuracy of the proposed diffuse interface approach. Moreover, additional numerical results on the same test are presented to demonstrate its mesh-independence feature, and, ultimately, to further demonstrate its reliability for the plain UHPFRC case.

The second part of the numerical outcomes (Section 5) is devoted to the application of the proposed integrated numerical framework (including the above-mentioned embedded truss model) to the case of steel bar-reinforced nano-enhanced UHPFRC structures. The main numerical outcomes, here presented in terms of both global structural response and final crack pattern, show the ability of the proposed approach to predict the load-carrying capacity of such structures, as well as to highlight the role of the embedded nano-reinforcement in the crack width control.

## 2. Diffuse Cohesive Interface Modeling Approach for Nano-Enhanced UHPFRC Structures

In this section, the general theoretical formulation of the diffuse cohesive interface approach for concrete-like materials is recalled, with reference to the planar elasticity case, and the particular mixed-mode interface model adopted for nano-enhanced UHPFRC is described.

### 2.1. General Cohesive Finite Element Formulation

The general theoretical formulation underlying the adopted diffuse cohesive interface approach for concrete-like materials is presented, following the variational framework described in [51]. This formulation refers to a general two-dimensional fractured body, occupying the region $\Omega \subset R^{2}$, subjected to body forces $\bar{f}$ in Ω and surface forces $\bar{t}$ on its Neumann boundary Γ*_N_*, and containing multiple discontinuity lines, which represent potential crack paths whose location is not known a priori. In this situation, all the internal mesh boundaries may be regarded as interfaces at which displacement jumps may occur, so that the variational formulation of the resulting equilibrium problem is written for a spatially discretized and split body.

**Figure 1:**
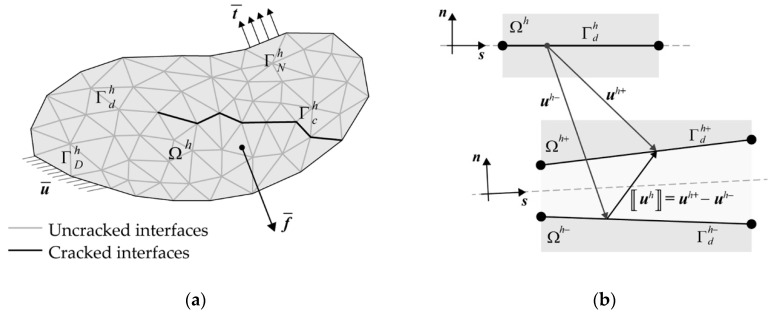
Equilibrium problem for a two-dimensional fractured body: (**a**) schematic representation of the spatially discretized body Ω*^h^*; (**b**) representation of the generic cohesive interface $\Gamma_{d}^{h}$

In detail, the original body Ω is replaced with its planar tessellation Ω*^h^*, depicted in Figure 1a, where initially undamaged cohesive interfaces, collectively denoted as $\Gamma_{d}^{h}$, are embedded along its internal boundaries, coinciding with all the potential crack sites. It follows that the actual crack pattern turns to be approximated by the subset of damaged cohesive segments, referred to as $\Gamma_{c}^{h}\subset \Gamma_{d}^{h}$, which are characterized by a nonlinear behavior according to a given softening law. The associated BVP expressed in weak form reads as follows: Find $u^{h}\in U^{h}$ such that:

(1) $$\int_{\Omega^{h}\backslash \Gamma_{d}^{h}}C\varepsilon \left(u^{h}\right)\cdot \varepsilon \left(v^{h}\right)d\Omega +\int_{\Gamma_{d}^{h}}K\left(d_{i}\right)〚u^{h}〛\cdot 〚v^{h}〛d\Gamma =\int_{\Omega^{h}\backslash \Gamma_{d}^{h}}\bar{f}\cdot v^{h}d\Omega +\int_{\Gamma_{N}^{h}}\bar{t}\cdot v^{h}d\Gamma \;\;\;\;\;\;\forall v^{h}\in V^{h}$$

where ***u****^h^* denotes the (unknown) approximated displacement field, *U^h^* is the set of kinematically admissible approximated displacement fields, ***v****^h^* is the (arbitrary) virtual displacement field, and *V^h^* is the set of kinematically admissible variations of the approximated displacement field. In addition, ε(•) is the usual linear strain operator, and ***C*** is the fourth-order elasticity tensor, whereas $〚•〛={•}^{+}-{•}^{-}$ is the jump operator across the interfaces $\Gamma_{d}^{h}$, and ***K*** denotes the second-order cohesive constitutive tensor, here assumed to be function of a finite set of scalar state (damage) variables *d_i_*, being responsible for the irreversible behavior of the embedded cohesive interfaces.

If not direct cross-coupling between normal and tangential deformation modes is assumed, ***K*** can be expressed in terms of normal and tangential cohesive stiffness terms, denoted as *K_n_* = *K_n_*(*d_i_*) and *K_s_* = *K_s_*(*d_i_*), respectively:

(2) $$K=K_{n}n⊗n+K_{s}\left(I-n⊗n\right)$$

where ***I*** denotes the second-order identity tensor, and ***n*** is the unit normal vector to $\Gamma_{d}^{h}$ (see Figure 1b). It follows that the embedded cohesive interfaces (which are of the so-called intrinsic type) behave as two beds of normal and tangential nonlinear springs, whose initial stiffness terms $K_{n}^{0}=K_{n}\left(d_{i}=0\right)$ and $K_{s}^{0}=K_{s}\left(d_{i}=0\right)$ play the role of penalty parameters (without having a precise physical meaning) to enforce the inter-element continuity of undamaged interfaces.

It is worth noting that, in concrete-like materials, being characterized by a quasi-brittle structural response with highly localized damage, only a small percentage of cohesive interfaces are cracked, whereas the remaining (undamaged) interfaces are worthless. It follows that the artificial compliance introduced by the elastic branch of these unnecessary interfaces negatively affects the mechanical response of the ideally uncracked body by seriously reducing its overall material stiffness. Such a compliance could be reduced by adopting sufficiently high interface stiffness parameters, and by considering, at the same time, some theoretical upper bounds for these parameters related to the need of avoiding any ill-conditioning of the tangent stiffness matrices in static analyses, as is well known in the literature [54,55,56].

In the present work, the normal and tangential stiffness parameters are assumed to be equal, and computed according to the following relation:

(3) $$K_{n}^{0}=K_{s}^{0}=K^{0}=\kappa \frac{E}{L_{\mathrm{mesh}}}$$

where *E* denotes the Young’s modulus of the bulk material, *L*_mesh_ is the average mesh size, and *κ* is a dimensionless stiffness parameter, imposed to be much greater than one to avoid any sensible artificial stiffness reduction. In particular, *κ* is set equal to 200 in this work. This value is coherent with what is suggested in the recent literature [52,57], and perfectly in line with an imposed reduction in the overall stiffness of about 1%, according to the micromechanical approach proposed by some of the authors to obtain invisible cohesive interfaces [51].

### 2.2. Traction–Separation Law for Nano-Enhanced UHPFRC Structures

In this section, the adopted traction–separation law (TSL) for nano-enhanced UHPFRC is presented. This is a trilinear softening model able to capture the tensile failure of fiber-reinforced concretes, initially proposed in [48] for functionally graded FRC and here adapted to the specific case of UHPFRC with embedded nanomaterials. In particular, all the microscopic fracture mechanisms of FRCs are taken into account, including cement paste micro-cracking, matrix/aggregate debonding, and fiber pull-out.

**Figure 2:**
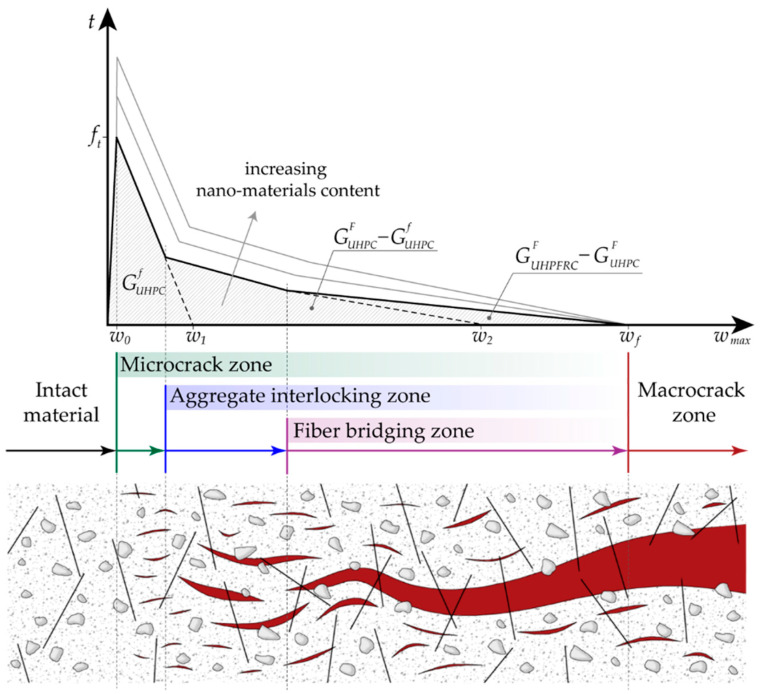
Traction–separation law for nano-enhanced UHPFRC with a trilinear softening model, and microscopic fracture mechanisms corresponding to each linear descending branch.

The adopted model also considers, in addition to the fracture resistance offered by the aggregate interlocking, the additional toughening effect of embedded discrete fibers, which is associated with a sensible enlargement of the fracture process zone size in (nano-enhanced) UHPFRC with respect to (nano-enhanced) UHPC without reinforcement. The first two linear descending branches of the adopted trilinear softening model for nano-enhanced UHPFRC are associated with the initial and total fracture energies of nano-enhanced UHPC, respectively, whereas the last descending slope is related to the additional energy required to debond and pull-out the fibers from the cement paste, corresponding to the difference between the total fracture energy of nano-enhanced UHPFRC and the total fracture energy of nano-enhanced UHPC, as shown in Figure 2.

The numerical calibration of this softening model requires the determination of the following six fracture parameters: tensile strength *f_t_*, initial fracture energy $G_{\mathrm{UHPC}}^{f}$ and total fracture energy $G_{\mathrm{UHPC}}^{F}$ of the plain (nano-enhanced) UHPC, critical crack tip opening displacement CTOD*_c_*, total fracture energy $G_{\mathrm{UHPFRC}}^{F}$ of (nano-enhanced) UHPFRC, and final crack opening width *w_f_*. The first four parameters refer to the bilinear softening model of the plain (nano-enhanced) UHPC, whereas the two latter ones define the last descending branch of nano-enhanced UHPFRC.

The horizontal axis intercepts of the first and second softening slopes are expressed as:

(4) $$w_{1}=\frac{2G_{\mathrm{UHPC}}^{f}}{f_{t}}$$

and

(5) $$w_{2}=\frac{\frac{2G_{\mathrm{UHPC}}^{F}}{f_{t}}-{\mathrm{CTOD}}_{c}}{1-\frac{{\mathrm{CTOD}}_{c}f_{t}}{2G_{\mathrm{UHPC}}^{f}}}$$

respectively, where the expression for *w*_2_ is derived assuming that the kink point between the first and second descending branches of the TSL is characterized by a crack opening width *w* (i.e., its abscissa in Figure 2) coinciding with the CTOD*_c_*, supposed to be a known material property (for additional details about the derivation of Equation (5), please see [58] and references cited therein). Finally, *w_f_* is estimated as *L_f_*/4, *L_f_* being the fiber length. Such a value corresponds to the estimated pull-out length for randomly distributed short fibers (see [48] for additional details), assumed to be insensitive to the amount of embedded nanomaterials, as clearly visible in Figure 2. This is realistic, since the scale involved in the final stage of pull-out is much greater than the nanoscale, affecting only the early stage of pull-out.

The associated complete mixed-mode intrinsic-type interface constitutive behavior, including the damage irreversibility and the frictionless unilateral contact conditions, reads as:

(6) $$\begin{array}{cc} t_{n}=\{\begin{array}{cc} K^{0}w_{n} & w_{n}<0\\ \frac{t\left(w_{\max}\right)}{w_{\max}}w_{n} & w_{n}\geq 0 \end{array}, & t_{s}=\frac{t\left(w_{\max}\right)}{w_{\max}}w_{s} \end{array}$$

where *w_n_* and *w_s_* denote the normal and tangential components of the displacement jump vector, the initial stiffness *K*^0^ is used to enforce in an approximated manner the non-interpenetration condition in compression (i.e., for *w_n_* < 0), and the function *t*(*w*_max_) represents the TSL shown in Figure 2, *t* being the effective traction and *w*_max_ the maximum value of the effective displacement jump *w* attained over the entire deformation history.

In this work, the same traction–separation law has been applied to both normal and nano-enhanced UHPFRC (with special reference to UHPFRC with different contents of embedded graphite nanoplatelets). The effect of diffuse nano-reinforcement on the nonlinear softening response of the conglomerate consists of a sensible increase of both tensile strength *f_t_* and fracture energies $G_{\mathrm{UHPC}}^{f}$, $G_{\mathrm{UHPC}}^{F}$ and $G_{\mathrm{UHPFRC}}^{F}$, as shown in Figure 2, due to the improved bond within the cement paste as well as at the cement paste/aggregate and cement paste/fiber interfaces. As a particular case, the resulting softening curve of normal UHPFRC (i.e., without nano-enhancement) is the lowest limit curve of the family depicted in the same Figure 2. Moreover, the final crack opening width *w_f_* is assumed to be insensitive to the incorporation of nanoplatelets within the concrete matrix and the fiber/matrix bond-slip behavior occurring at higher spatial scales, which are not interacting with the nanoscale level.

Finally, it is worth noting that the above-described interface model is not suitable for hooked fibers and/or high fiber volume fractions, which are usually associated with a strain hardening behavior in the post-cracking stage, even accompanied with a secondary peak in the global traction–separation response [49,50,59]. However, such a behavior is not considered here, being outside of the scopes of the present work, but could be the object of future investigations involving a wider class of UHPFRCs.

## 3. An Integrated Numerical Model for UHPFRC Structures Enhanced with Nanomaterials

This section is devoted to the presentation of the newly proposed numerical framework for the failure analysis of nano-enhanced UHPFRC structures, as well as of the related computational details. Such a framework is adapted from another integrated numerical model, previously introduced in [53] for conventional RC structures, to the case of steel bar-reinforced nano-enhanced UHPFRC structures.

This numerical model is composed of two distinct submodels: (a) a diffuse interface model (DIM) for the simulation of multiple cracking within the concrete phase, introduced in [51]; and (b) an embedded truss model (ETM) able to simulate the yielding behavior of steel reinforcing bars, as well as their interaction with the surrounding concrete layer, by virtue of a suitably calibrated bond–slip interface model. The synergistic combination of these two submodels allows the potential damage mechanisms in steel bar-reinforced nano-enhanced UHPFRC elements to be accurately predicted, including flexural/shear cracking and steel/concrete bond failure.

In particular, the adopted model is able to correctly simulate the so-called tension stiffening effect, which is related to the capability of sound concrete to sustain tensile stresses between adjacent primary cracks. To this end, the steel/concrete interface model plays a fundamental role in predicting the structural behavior of steel-bar reinforced UHPFRC members in their cracked stage, since the steel/concrete slip behavior strongly influences the distribution of bond stresses along the reinforcement bars, and, ultimately, both crack width and crack spacing values.

**Figure 3:**
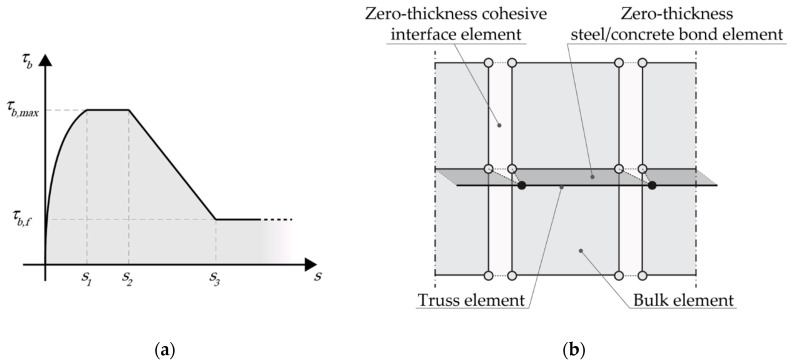
Representation of the steel/concrete interface model: (**a**) bond stress–slip constitutive behavior taken from CEB-FIP Model Code for Concrete Structures 2010 [61]; (**b**) schematic of embedded truss elements and zero-thickness steel/concrete bond elements.

According to the adopted ETM, the reinforcing bars are modeled as elastic-plastic truss elements, which are connected to the concrete elements via special zero-thickness interface elements equipped with a bond-slip constitutive behavior, able to capture the steel/concrete interactions (see Figure 3a). The bond stress–slip relation proposed in [60] is adopted, which is a modification of that proposed by CEB-FIP Model Code 2010 [61], obtained by assuming, for (nano-enhanced) UHPFRC, $\tau_{b,\max}=3.9\sqrt{f_{c}}$ (*f_c_* being its mean compressive strength), *s*_1_ = 0.1 mm and *s*_2_ = 0.6 mm. The remaining parameters are taken from the original Model Code formula; thus, *τ_b_*_,*f*_ = 0.4*τ_b_*_,max_ and *s*_3_ = 10 mm, coinciding with the distance between ribs. The interface behavior is completed by assuming a perfect steel/concrete bond in the normal direction, so that only the interfacial slip is regarded as an active degree of freedom.

It is worth noting that the influence of nano-reinforcement on the adopted bond-slip relation for nano-enhanced UHPFRCs is implicitly taken into account by assigning a variation to *f_c_* depending on the content of the embedded nanomaterials, which corresponds to a modification of the maximum steel/concrete bond stress. In more detail, according to the adopted model, an increase in the content of embedded nanomaterials, and thus in the compressive strength of the resulting conglomerate, is associated with an increase in the bond strength with the interacting steel bars. In the present work, different mean compressive strength values for different nano-enhanced UHPC mixtures have been directly taken from the literature, as will be explained in Section 5.1.

A fundamental aspect of the adopted ETM relies on the possibility for any crack propagating towards the reinforcing bars to cross the steel layer, as depicted in Figure 3b, thus avoiding artificial propagation constraints or, even worse, crack arrests, related to the 2D representation of the model. According to this model, the crack width at each steel node is computed as the sum of the two independent slip values related to the adjacent steel/concrete interface elements.

The proposed integrated numerical framework for the failure analysis of UHPFRC structures (with or without nano-enhancement) has been implemented within the commercial finite element environment COMSOL Multiphysics [62], chosen for its advanced scripting capabilities, including the possibility to easily develop and apply user-defined cohesive interface elements.

## 4. Numerical Application to Plain Nano-Enhanced UHPFRC

In this section, the main numerical results obtained via the adopted diffuse cohesive interface model for plain nano-enhanced UHPFRC are presented, with reference to a simulated flexural test which involves structural elements containing different volume fractions of graphite nanoplatelets (GNPs). The proposed numerical applications have the twofold role of calibrating the inelastic parameters of embedded interfaces and of assessing the numerical accuracy of the adopted fracture model, via suitable comparisons with available experimental results, shown in Section 4.1. Furthermore, in Section 4.2, additional numerical computations are presented, aimed at demonstrating the mesh size independency of the predicted nonlinear structural response.

### 4.1. Model Calibration and Comparison with Experiments

**Figure 4:**
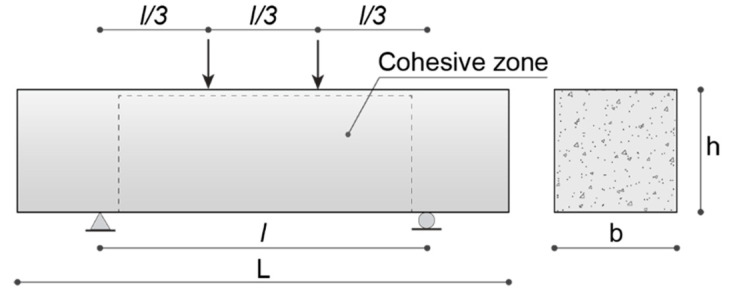
UHPFRC beam geometry and boundary conditions of the four-point bending test.

The present numerical simulations involve small-sized UHPFRC beam specimens subjected to a four-point bending test, analyzed in [33] from an experimental point of view, whose geometry and boundary conditions are depicted in Figure 4.

The geometric parameters of the cross section are *b* = 76 mm and *h* = 76 mm, whereas the total length and the span length of the beam are equal to *L* = 305 mm and *l* = 203 mm, respectively. Three mixtures of UHPFRC, containing 0.5% by volume of straight steel fibers with length *L_f_* = 13 mm and diameter *D* = 2 mm, have been used for the specimens considered in the next simulations: one mixture without nano-enhancement, which is taken as the control one and named as UHPFRC, and two mixtures containing 0.05% and 0.1% of GNP reinforcements, referred to as UHPFRC GNP 0.05% and UHPFRC GNP 0.1%, respectively. Both physical and mechanical properties of the GNP and steel fiber reinforcement are reported in Table 1.

**Figure 5:**
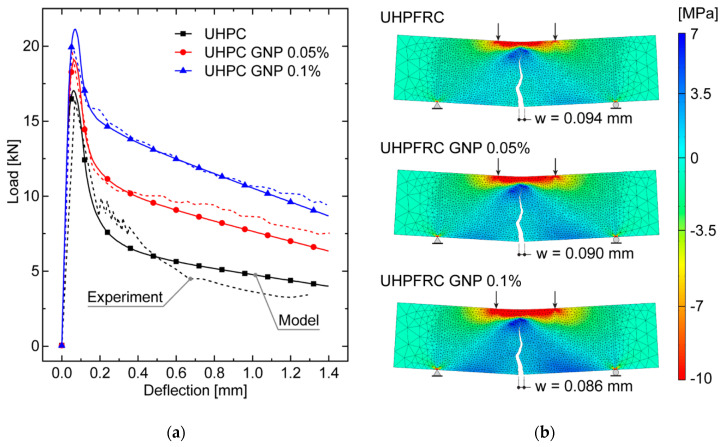
Global structural response for the three considered UHPFRC mixtures: (**a**) comparison between numerical and experimental results in terms of load versus mid-span deflection curves; (**b**) deformed configurations (magnified by a scale factor of 25), horizontal stress maps and main crack paths at a beam deflection of 0.2 mm.

The elastic bulk material parameters have been set as equal for all the three concrete mixtures, being assumed to be almost independent of GNP embedding at lower volume fractions. In particular, the adopted Young’s modulus is *E* = 40 GPa, taken from the uniaxial tensile test results reported in [33], whereas the Poisson’s ratio is *ν* = 0.2, as usually assumed for uncracked normal concretes. The adopted values of the inelastic constitutive parameters introduced in Section 2.2 are shown in Table 2 for all the considered mixtures. The tensile strength values *f_t_* are directly taken from the uniaxial tensile test responses reported in [33], whereas the fracture energies $G_{\mathrm{UHPC}}^{f}$, $G_{\mathrm{UHPC}}^{F}$, and $G_{\mathrm{UHPFRC}}^{F}$ as well as the CTOD*_c_* values are obtained by means of a fitting procedure of the experimental load–displacement curves obtained for the four-point bending test analyzed in [33] and reported in Figure 5 for comparison purposes. As constraints introduced to simplify the calibration procedure, the CTOD*_c_* is assumed to be constant for all the mixtures, and the fracture energy values are adjusted to enforce a linear variation with the nanoplatelet content. The constant value for the CTOD*_c_* is not intended as an exact value (which should coincide with the real crack tip opening displacement at the original crack tip of the specimen using the measured ultimate load level for each GNP content), but rather can be regarded as an average with respect to the considered range of variation for the GNP content. As expected, the estimated fracture energies $G_{\mathrm{UHPC}}^{f}$, $G_{\mathrm{UHPC}}^{F}$, and $G_{\mathrm{UHPFRC}}^{F}$ increase for increasing values of the nanoplatelet volume fraction (at least within the considered range of variations). It is worth noting that these fracture energy values are intended not to be valid in general, but rather as reasonable estimates, providing enough accurate predictions for the investigated cases.

To reduce the computational effort of the numerical analyses, the cohesive interface elements have been inserted only within the rectangular area highlighted in Figure 4, which is dominated by a combined tension–shear stress state. Here, a suitably refined triangular tessellation has been generated by using an isotropic Delaunay algorithm and imposing a maximum element size of 4 mm, which corresponds to an average length of interface elements of about 2.93 mm. The resulting mesh is composed of 3890 three-node bulk elements and 5903 four-node zero-thickness interface elements. The subsequent numerical computations have been conducted under quasi-static loading conditions via a displacement-controlled path-following scheme, by adopting a constant vertical displacement increment of 5 × 10^−3^ mm for the extrados point of the mid-span section. Moreover, all the numerical simulations have been performed under a plane stress assumption.

The structural responses numerically derived for the different UHPFRC mixtures, depicted in Figure 5a, have been compared with the experimental results reported in [1]. The load versus mid-span deflection curves, obtained by exploiting the diffuse cohesive interface modeling approach presented in Section 2, can be schematized with three clearly detectable branches for each concrete mixture: the first one corresponds to the elastic regime in which the load level increases almost linearly up to the nucleation of the main crack, occurring at the mid-span section due to the symmetric geometry and boundary conditions. After the peak load, which is associated with the early stage of crack propagation, a fast crack growth characterizes the second branch of the numerically derived curve, along which the load level decrease until the main crack arrest occurs, due to the steel fiber bridging effect. Then, in the final part of the softening branch, owing to the activation of the fiber pull-put forces, the load level does not drop to zero but remains at (slowly decreasing) residual values.

It is worth noting that the adopted diffuse cohesive methodology allows both nucleation and propagation of the main crack to be naturally predicted, as shown in Figure 5b, without assuming the preexistence of weak zones or requiring the introduction of an initial stress-free crack. The potential drawback of this methodology consists of the resulting mesh-dependency of the predicted crack path, which will be explicitly investigated in more detail by a suitable sensitivity analysis, presented in the following Section 4.2.

The good agreement between the experimental and numerical loading curves for each investigated UHPFRC mixture underlines that the adopted traction–separation law, if suitably calibrated in terms of inelastic cohesive parameters, is reliable for determining the structural behavior of both normal and nano-enhanced UHPFRC elements with lower nanoparticle contents (up to 0.1% by volume). The percentage errors on the predicted load peak with respect to the experimental values are fully acceptable from an engineering point of view, being of 3.62%, 3.58%, and 4.51%, for the three mixtures UHPFRC, UHPFRC GNP 0.05%, and UHPFRC GNP 0.1%, respectively. Moreover, a slight local divergence between numerical and experimental results can be observed in the softening branch of the curves referring to the case of UHPFRC without nano-reinforcement, probably due to the occurrence of an unstable structural response in the experimental test, being associated with the appearance of dynamic effects (totally neglected in the numerical simulations), characterized by very high speeds of the main crack propagation.

The numerical outcomes clearly demonstrate the ability of the proposed diffuse cohesive model for UHPFRC in capturing the effectiveness of the embedded reinforcement in the form of graphite nanoplatelets on the mechanical performances of small-scale structural elements, in terms of cracking resistance and fracture toughness. As a matter of fact, as known from the experiments and confirmed by numerical results, an increase in the GNP fraction leads to an increase of both the peak load and the energy absorption capacity, due to the increase in the bond strength between cement paste and steel fibers and between cement paste and fine aggregates, guaranteed by the additional work-of-fracture provided by embedded nanoplatelets. On the other hand, as illustrated in Figure 5b, in the presence of nano-enhancements, significant beneficial effects can be observed in terms of more controlled crack patterns, associated with an increased apparent ductility in the post-peak stage. Indeed, the inclusion of graphite nanoplatelets in the concrete matrix inhibits the nucleation of micro-cracks, reducing at the same time the (macroscopic) crack width. In particular, percentage reductions in the main crack width of about 4% and 8% are obtained for UHPFRC GNP 0.05% and 0.1% cases, respectively, both compared to the control UHPFRC mixture (without nano-reinforcement).

### 4.2. Mesh Size Sensitivity Analysis

With the purpose of investigating the mesh influence on the fracture behavior predicted by the diffuse interface model, a sensitivity analysis is performed by varying the mesh size, with reference to the already examined control UHPFRC specimen (i.e., without nano-enhancement). Both the elastic and inelastic properties of cohesive interfaces are those reported in Table 2 for the first UHPFRC mixture, and the geometry and loading conditions are those considered in the previously simulated test. Four Delaunay meshes have been built considering different refinements within the region susceptible to damage detected in the previous section, obtained by dividing in half the maximum element size from 16 to 2 mm. The resulting meshes, numbered from 1 (the coarsest mesh) to 4 (the finest mesh), are characterized by an average element size of about 11.9, 5.95, 2.93, and 1.45 mm, corresponding to a number of interface elements of 372, 1454, 5903, and 23,463, respectively.

**Figure 6:**
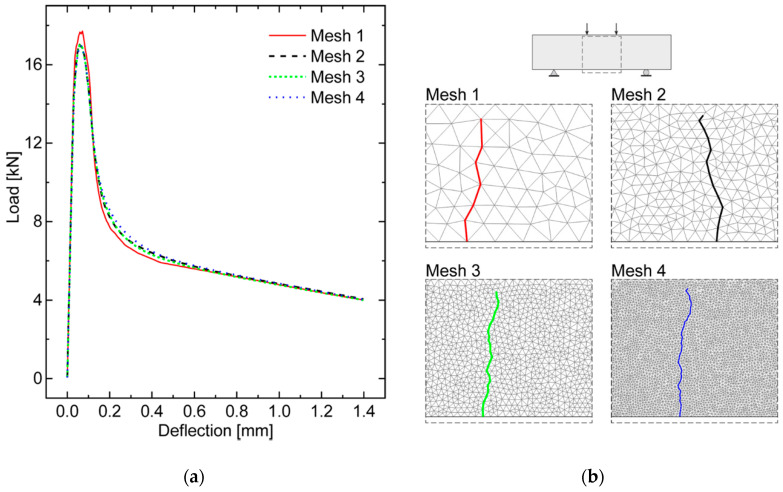
Global structural response of the control UHPFRC beam for different mesh sizes: (**a**) load versus mid-span deflection curve; (**b**) main flexural crack path within the cohesive region.

The numerical analyses show that the global structural response in terms of load versus mid-span deflection curve, depicted in Figure 6a, is almost independent of the mesh size. Some convergence troubles, especially on the peak load, have been observed by the simulation performed with the Mesh 1, probably due to the fact that the adopted discretization is not sufficiently refined to evaluate in an accurate manner the stress gradients within the fracture process zone as well as along the uncracked ligament ahead of the (moving) crack tip. On the contrary, the other three meshes provide almost coincident numerical results in terms of global structural response. In particular, the percentage error on the peak load with respect to the finest mesh case (i.e., Mesh 4), computed as:

(7) $$e_{P,i}=\frac{\left|P_{i}^{\max}-P_{4}^{\max}\right|}{P_{4}^{\max}}\times 100\;\;\;\;\;\;\;i=2,3$$

for each investigated mesh (excluding Mesh 1), presents a maximum value of about 0.71%, which corresponds to Mesh 2. This further outcome confirms the desired mesh-independency property of the adopted diffuse cohesive model.

Finally, an investigation on the mesh sensitivity of the numerically predicted main crack path is illustrated in Figure 6b. As excepted, owing to the randomness of the generated meshes, a loss of convergence is experienced in terms of predicted crack path. In particular, the casual distribution of the cohesive interface elements at the lower side of the beam inevitably leads to different crack onset locations and, as a consequence, to different crack propagation path segments for the different tessellations, being forced to lie along different mesh boundaries.

Nevertheless, despite the above-mentioned local deviations, the predicted main crack at the end of simulation has been found to be almost insensitive to the mesh in a global sense, being always directed along nearly vertical directions and comprised within the constant bending moment region, with an average trajectory located in the proximity of the mid-span section (coinciding with the theoretically predicted crack path due to the initial symmetry conditions existing in the test setup). Such an alleviated mesh dependency behavior is essentially found by virtue of the adoption of a Delaunay triangulation, which preserves the isotropic nature of fracture processes in concrete-like materials, without introducing any preferential crack path directions with no physical meaning.

The above results can be regarded as a further validation step of the diffuse interface model proposed in [51] and, more generally, confirm the reliability of the adopted numerical framework to predict the overall structural response of UHPFRC structures.

## 5. Numerical Application to Steel Bar-Reinforced Nano-Enhanced UHPFRC

In this section, the load-carrying capacity of steel bar-reinforced UHPFRC elements enhanced with graphite nanoplatelets (GNPs) has been investigated by means of the numerical model described in Section 3. Such a model is given by the combination of a diffuse interface model (DIM) and an embedded truss model (ETM), able to capture the diffuse damage processes which are typical of UHPFRC structures, and to simulate the steel/concrete interactions, respectively.

The subsequent numerical analyses have been performed to evaluate the structural response of the same steel bar-reinforced UHPFRC beam configuration with and without GNP reinforcements. The related results in terms of global load-deflection curves, horizontal stress maps, and tensile stress trends along the lower reinforcement bars are reported in Section 5.2.

### 5.1. Geometric and Material Properties

**Figure 7:**
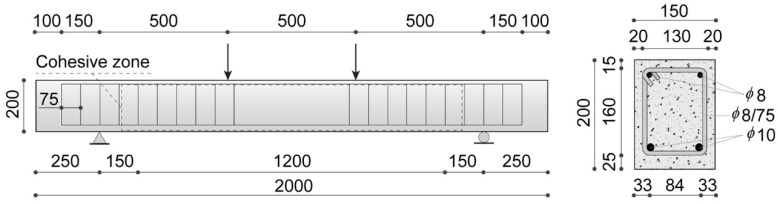
Geometric configuration, loading conditions and constrains of the simulated four-point bending test (all dimensions are expressed in mm).

The numerical application of the proposed integrated framework for steel bar-reinforced UHPFRC consists of the simulation of four-point bending tests performed on different medium-sized steel bar-reinforced beams made with the UHPFRC mixtures already adopted in Section 4, characterized by the same steel fiber content and geometry, and three different pieces of GNP content (0%, 0.05%, and 0.1%). The related geometric configuration, loading conditions, and constraints, shown in Figure 7, are taken from the experimental tests performed on normal RC beams found in [63], and already considered by some of the authors as reference data for the numerical simulation of concrete cover separation in RC structures [52]. Figure 7 also shows all the dimensions of both longitudinal bars and stirrups. It is worth noting that no stirrups are present in the beam region comprised between the two applied concentrated loads, being characterized by the absence of average shear stresses.

The main mechanical parameters of both UHPFRC and steel materials are listed in Table 3. In particular, the UHPFRC elastic properties are the same as those considered in Section 4.1, whereas the UHPFRC compressive strength, required for the computation of the maximum concrete/steel shear stress according to the bond-slip model described in Section 3, is directly taken from [33].

The computational mesh, consisting of 17,346 triangular elements with a maximum element size of 10 mm, has been generated by using a Delaunay triangulation algorithm to avoid any preferential crack path direction. As shown in Figure 7, to preserve a significant computational efficiency, the extension of the cohesive insertion zone has been limited to the region comprised between the supports, being the only one susceptible to being cracked due to the presence of completely free boundary conditions outside of this region. Moreover, the embedded cohesive elements lying along the steel reinforcements are excluded from the numerical model. This latter expedient has been adopted to prevent the onset of fractures along preferential paths identified by the horizontal and vertical straight lines coinciding with the longitudinal bars and stirrups, respectively. The inelastic cohesive parameters required by the adopted interface constitutive law are the same as those used in the previous numerical application and listed in Table 2, being already calibrated in Section 4.1 for the considered UHPFRC mixtures.

Finally, all the steel reinforcements (i.e., both longitudinal bars and stirrups) are modeled as one-dimensional two-node elastic-plastic truss elements connected to concrete elements via special zero-thickness four-node bond elements, according to the embedded truss model sketched in Figure 3b. The following numerical simulations have been performed under plane stress and quasi-static assumptions, adopting a displacement-control solution scheme with constant increments of the mid-span deflection equal to 5 × 10^−2^ mm.

### 5.2. Numerical Results and Discussion

**Figure 8:**
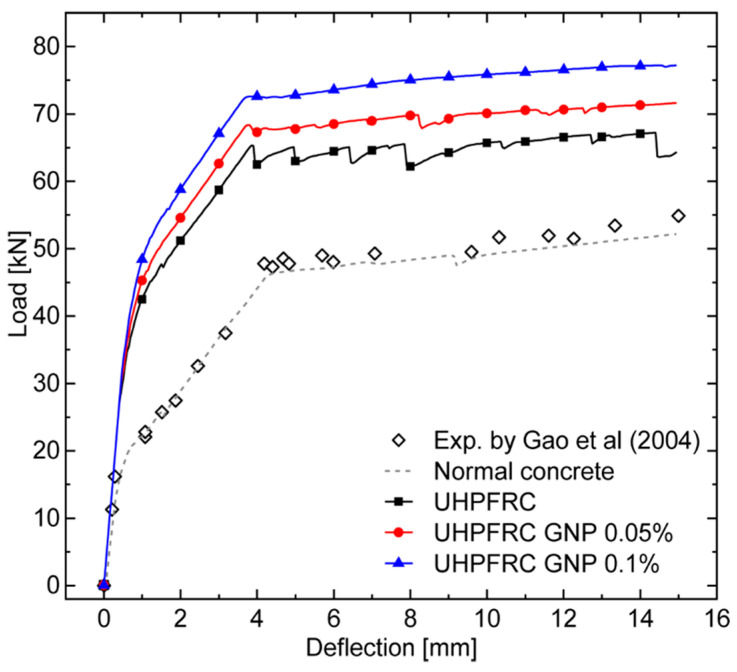
Numerically predicted load versus mid-span deflection curves of steel bar-reinforced UHPFRC beams enhanced with different content of GNPs (0%, 0.05%, and 0.1%).

This section is devoted to the presentation and subsequent discussion of the numerically predicted structural response of the considered steel bar-reinforced GNP-enhanced UHPFRC beams. Such a response in terms of total load versus mid-span deflection curves is reported in Figure 8 for all the three concrete mixtures. The total load is measured as the sum of applied concentrated forces on the upper side of the beams.

For comparison purposes, this figure also shows the experimental results reported in [63], represented as scattered points, together with the related numerical results obtained for a normal concrete (i.e., without steel fibers), represented by a dotted line. The latter results have been derived by performing an additional simulation introduced only for verification purposes. To this end, the trilinear softening model discussed in Section 2.2 has been adapted to normal concrete, neglecting the third descending branch of Figure 2 and assuming the following concrete properties, coherent with the well-known softening model proposed by Petersson [64]: Young’s modulus *E* = 31 GPa, Poisson’s ratio *ν* = 0.2, tensile strength *f_t_* = 2.1 MPa, initial fracture energy *G_f_* = 75 N/m, total fracture energy *G_F_* = 125 N/m, and critical crack tip opening displacement CTOD*c* = 0.048 mm. The excellent agreement between the experimental and numerical results further confirms the reliability of the proposed numerical framework for the failure prediction of both RC and FRC structural elements.

As expected, the loading curves referring to steel bar-reinforced UHPFRC beams (with and without GNP enhancement) show greater load-carrying capacities with respect to the conventional RC beam, with increasing strength values for increasing contents of embedded graphite nanoplatelets (from 0% to 0.1%).

In particular, the typical trilinear behavior of steel bar-reinforced structural elements has been observed for all the analyzed cases. The first slope change coincides with the occurrence of early nonlinear phenomena, consisting of coalescence of concrete microcracks and subsequent macrocrack nucleation. After this, the second linear branch with reduced stiffness initiates after the crack saturation state is reached, being associated with multiple macrocrack propagation toward the upper side of the RC beam. Finally, the second slope change corresponds to the initiation of the yielding phase for lower steel rebars, after which a slight hardening is kept without the occurrence of any collapse until the end of simulation, stopped as the deflection reaches a prescribed value of 15 mm.

The numerical results clearly show that the combination of micro- and nano-reinforcements (in the form of steel fibers and graphite platelets, respectively) significantly improves the flexural behavior of UHPFRC beams, in terms of ultimate load and energy absorption. In particular, increments of 11% and 20% in the absorbed energy (computed as the area under each load-displacement curve reported in Figure 8), as well as increments of 4.8% and 11% in the first yielding load level, with respect to the UHPFRC case, are reached with contents of nano-reinforcement equal to 0.05% and 0.1%, respectively. Such an increased strength at both peak and post-peak stages is essentially due to the concurrence of two phenomena. The first one consists of a stronger crack bridging effect of steel microfibers promoted by the high reactivity of embedded interacting nanomaterials, which allows macrocrack propagation to be retarded, thus leading ultimately to a stronger bond between steel reinforcing bars and surrounding concrete (see, for instance, [65] and references cited therein). The second one is the additional crack bridging effect at the nanoscopic scale within the cement paste, responsible for an increase in the tensile strength of UHPFRC material.

Both phenomena, considered individually and/or in synergy, contribute to amplifying the significance of the well-known tension stiffening effect characterizing the interaction between steel reinforcing bars and surrounding concrete layers.

**Figure 9:**
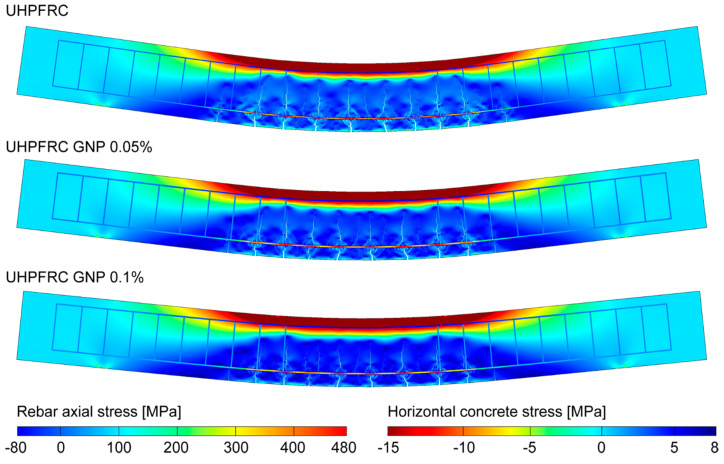
Deformed configurations (magnified by a multiplicative factor equal to 15) and stress maps for the three simulated steel bar-reinforced GNP-enhanced UHFRC beams at a load level of 65 kN.

The role of GNPs on the tension stiffening effect can be better highlighted by analyzing the numerically predicted cracking patterns clearly visible in the deformed configurations reported in Figure 9, as obtained for the three investigated concrete mixtures at the same load level of 65 kN (corresponding to the first yielding of tensile reinforcing bars of the UHPFRC beam without GNPs). It is worth noting that, owing to the higher fracture toughening effect provided by the embedded nano-reinforcement, a significant reduction in the crack pattern development for the concrete mixture with the highest GNP volume fraction is experienced, compared to the other cases.

The analysis of crack patterns has been more deeply investigated by computing the crack spacing, average crack width, and beam deflection for different contents of GNP reinforcement, as reported in Table 4. It can be noted that the embedding of GNPs in the concrete mixture provides a sensible decrease in all the considered quantities. 

Specifically, by restricting the cracking analysis within the constant bending moment region, the average crack width values of 0.079 mm and 0.063 mm can be measured for the cases with GNP addition of 0.05% and 0.1%, respectively, corresponding to crack width reductions of 14% and 31%, respectively, compared to the case of UHPFRC without nano-enhancement (exhibiting an average crack width of 0.093 mm). In addition, the crack spacing for the three different mixtures has been measured at the same fixed load level of 65 kN, obtaining a mean value of 91 mm for the case without nano-enhancement and of 84 mm for both the nano-enhanced mixtures. A crack spacing reduction of 7.32% has been achieved with the introduction of nano-enhancement, while a non-relevant crack spacing reduction is observed for a GNP volume fraction equal to 0.1%, due to the fact that, for the analyzed configuration, such content of nano-reinforcement is associated with a diffuse micro-cracking within the concrete teeth between existing macro-cracks, without leading to the onset of new macro-cracks. Moreover, compared to the case without nano-enhancement, a beam deflection reduction equal to 13.2% and 27.7% has been achieved for the cases with GNP addition of 0.05% and 0.1%, respectively, highlighting an improvement of the overall mechanical performances in terms of increasing bending stiffness as the nano-reinforcement content increases.

**Figure 10:**
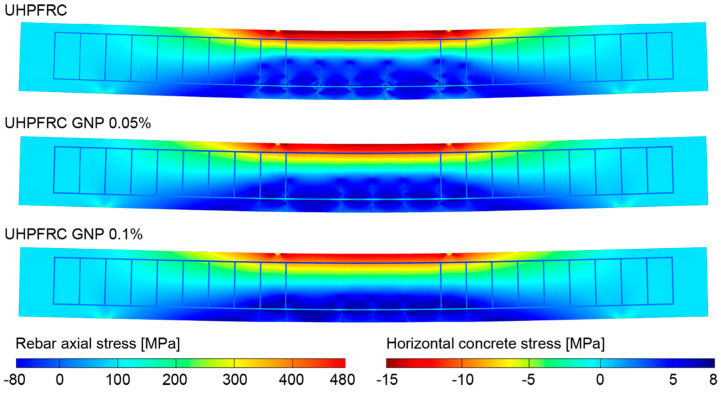
Deformed configurations (magnified by a multiplicative factor equal to 15) and stress maps for the three simulated steel bar-reinforced GNP-enhanced UHFRC beams at a load level of 45 kN.

With the aim to investigate the cracking phenomenon under service conditions, in Figure 10, the deformed beam configurations for the three investigated concrete mixtures have been reported at a load level of 45 kN, corresponding to the early stage of crack propagation at the bottom of the beams. Moreover, the average crack width, crack spacing and beam deflection at the same load level have been reported in Table 5.

In Figure 10, it can be seen that the cracking patterns are not completely developed, compared to those obtained at a load level of 65 kN, the further cracks being visible in Figure 9 in an incipient propagation stage at service conditions. More specifically, the crack spacing at service conditions, as reported in Table 5, is not influenced by the presence of nano-reinforcements, resulting in being equal to 100 mm for all the investigated mixtures. Contrary to what happens for the crack spacing, the average crack width is strongly influenced by the nano-reinforcement, which results in being equal to 0.019 mm and 0.012 mm for the cases with GNP addition of 0.05% and 0.1%, respectively, corresponding to a crack width reduction, compared to the case without nano-reinforcement, equal to 30.1% and 55.7%, respectively. The obtained crack width reduction at service condition reached by the addition of GNP results in being almost doubled compared to the one reached at a load level equal to 65 kN, highlighting a better fracture toughening effect provided by nano-reinforcements at service conditions. In addition, in Table 5, the beam deflection at service conditions has been also reported for the three investigated mixtures, highlighting a beam deflection reduction equal to 16.7% and 29.2% for the cases with GNP addition of 0.05% and 0.1%, respectively, compared to the case without nano-enhancement. As a consequence, not relevant changes in the beam deflection reduction have been observed compared to those evaluated at a load level equal to 65 kN, and, generally speaking, also at service conditions, the bending stiffness increases as the nano-reinforcement content increases. 

The numerically predicted crack width and crack spacing reductions for GNP-enhanced concrete highlights the reliability of the proposed numerical framework for UHPFRCs in capturing the additional crack bridging effect provided by nanoparticles inserted into the concrete mixture, and, ultimately, its beneficial influence on the ductility properties of structural elements, being intimately related to their macro-cracking behavior.

**Figure 11:**
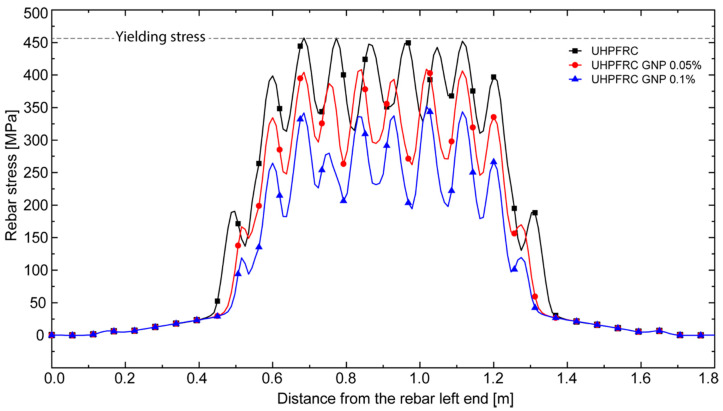
Axial stress distribution along the tensile longitudinal reinforcement bars of the three considered UHFRC beams with different contents of GNPs, for a load level of 65 kN.

Furthermore, in Figure 11, the axial stress distribution along the tensile reinforcement bars of the three simulated UHPFRC beams has been reported for the same load level (65 kN). The reported trends, characterized by lower values of the average rebar stress (and strain) for higher values of the GNP content, confirm the increase in the tension stiffening effect for increasing fractions of nano-reinforcement (at least within the considered range of variation). In particular, the reductions in the (global) maximum stress associated with 0.05% and 0.1% of GNPs with respect to the UHPFRC without nano-enhancement are of about 11% and 23%, respectively.

Finally, the reported oscillating behavior of such stresses, with local maxima in proximity of fully developed cracks and local minima between two contiguous cracks, demonstrates the capability of the adopted embedded truss model as well as of the proposed steel/UHPFRC bond-slip model to correctly capture the stress transfer between steel and concrete phases, which is of fundamental importance for the accurate numerical simulation of tension stiffening phenomena.

All of the obtained numerical results confirm the reliability of the proposed integrated numerical framework for the failure analysis of steel bar-reinforced nano-enhanced UHPFRC structures, but a complete validation of the present model is out of the scope of the present work, due to the limited amount of experimental data available in the literature, to the best knowledge of the authors. A more rigorous calibration of this model could be the object of future investigations, which could also be addressed for the development of a more general validation procedure, eventually involving additional experimental research activity on nano-enhanced UHPFRC structural elements.

## 6. Conclusions

In this work, an integrated numerical model relying on a diffuse cohesive interface approach is developed for tracing the structural response of steel bar-reinforced UHPFRC structures enhanced with nanomaterials. The main advantages of this model can be synthetized in the following points:

- possibility to accurately predict multiple crack initiation and propagation phenomena within a rather standard and readily implementable displacement-type finite element setting;
- capability to preserve the discrete nature of fracture processes, and, therefore, to capture the real complex crack patterns, in terms of both crack spacing and crack widths, whose knowledge is of fundamental importance for the safety and serviceability assessment of UHPFRC structures, as well as for their fiber content optimization within the design process.

In the first part of this work, a diffuse interface model for the failure analysis of UHPFRC has been applied to investigate the role of the content of embedded graphite nanoplatelets (GNPs) on its load-carrying capacity at both peak and post-peak stages, with reference to simply supported beams subjected to a four-point bending test. All the numerical outcomes have been validated by performing suitable comparisons with the available experimental results. A good accordance between numerical and experimental loading curves has been found, with a mean absolute percentage error on the peak load of only about 4%, by virtue of a proper calibration of the inelastic parameters of the embedded cohesive interfaces. Moreover, additional computations have been performed to investigate the mesh dependency effects on the global structural response and crack pattern. The related results have shown that, despite the (local) mesh-dependency of the numerically predicted crack paths, which is related to the unavoidable randomness in the mesh generation procedures, the (global) load–displacement curves are substantially independent of the adopted discretization.

In the second part, the proposed integrated numerical model for UHPFRC, which incorporates the adopted diffuse interface model, has been applied to investigate the effect of nano-enhancement in steel bar-reinforced UHPFRC structures. To this end, a simulated four-point bending test on three medium-sized beams with different volume fractions of GNPs is considered. The numerical outcomes have demonstrated the reliability and the accuracy of the proposed model in predicting both the strengthening and toughening effects of embedded nanomaterials, in terms of global load–deflection responses and associated crack patterns. In particular, increases in the first yielding load level and absorbed energy up to 11% and 20%, respectively, are numerically predicted for hybrid micro/nano-reinforcements with the highest considered GNP content (i.e., 0.1% by volume). Furthermore, the role of nanomaterials on the tension stiffening effect has been demonstrated, by analyzing both final crack patterns and associated stress distribution maps. From the numerical analyses, the addition of 0.05% and 0.1% of GNPs has led to crack width reductions of 14% and 31%, respectively, as well as to maximum axial stress reductions along steel rebars of about 11% and 23%, respectively, thus confirming the increased ductility of the enhanced UHPFRC, at least for small volume contents of nano-reinforcement.

As a possible future perspective of this research, the proposed numerical framework for the failure analysis of nano-enhanced UHPFRC structures could be incorporated within a more general and cost-effective computational strategy involving (eventually adaptive) multiscale methodologies similar to those already proposed by some of the authors for lightweight aggregate concretes [66], fiber- and particle-reinforced composite materials [67,68,69], and regular masonry structures [70]. Such an envisaged computational strategy could be used to find the optimal combination of micro- and nano-reinforcement to achieve a precise crack control, with the final aim of developing new highly durable UHPFRC mixtures with tailored properties, such as high fracture toughness and reduced permeability to water and aggressive chemicals.

## Figures and Tables

**Table 1:** Mechanical and physical properties of the GNP and steel fiber reinforcement (taken from [33]).

	Specific Gravity [g/cm^3^]	Elastic Modulus [GPa]	Tensile Strength [MPa]	Dimensions
GNP	1.95	1000	5000	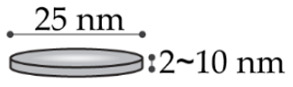
Straight steel fiber	7.85	203	1900	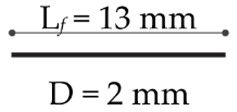

**Table 2:** Inelastic parameters of embedded cohesive interfaces for the three UHPFRC mixtures.

	$f_{t}\;$[MPa]	$G_{UHPC}^{f}$[N/m]	$G_{UHPC}^{F}\;[N/m]$	$G_{UHPFRC}^{F}\;[N/m]$	$CTOD_{c}\;[\mathrm{mm}]$	$w_{f}$ [mm]
UHPFRC	5.71 ^1^	40	350	1800	0.005	3.25
UHPFRC GNP 0.05%	6.14 ^1^	50	375	2800	0.005	3.25
UHPFRC GNP 0.1%	6.81 ^1^	60	400	3800	0.005	3.25

^1^ Values taken from the uniaxial tensile test reported in [33].

**Table 3:** Main mechanical properties of UHPFRC and steel materials.

	Young’s Modulus [GPa]	Poisson’s Ratio [-]	Yield Strength [MPa]	Tangent Modulus [GPa]	Compressive Strength [MPa]
Steel	200	0.3	460	2.0	-
UHPRFC	40	0.2	-	-	174
UHPRFC GNP 0.05%	40	0.2	-	-	176
UHPRFC GNP 0.1%	40	0.2	-	-	178

**Table 4:** Average crack width, crack spacing, and beam deflection of the simulated beams at a load level of 65 kN.

	Average Crack Width [mm]	Crack Spacing [mm]	Beam Deflection [mm]
UHPFRC	0.093	91	3.80
UHPFRC GNP 0.05%	0.080	84	3.30
UHPFRC GNP 0.1%	0.064	84	2.75

**Table 5:** Average crack width, crack spacing, and deflection of the simulated beams at a load level of 45 kN.

	Average Crack Width [mm]	Crack Spacing [mm]	Beam Deflection [mm]
UHPFRC	0.027	100	1.20
UHPFRC GNP 0.05%	0.019	100	1.00
UHPFRC GNP 0.1%	0.012	100	0.85
